# Peculiar bond characters of fivefold coordinated octet compound crystals†

**DOI:** 10.1039/d0sc00292e

**Published:** 2020-03-27

**Authors:** An-An Sun, Shang-Peng Gao, Gong Gu

**Affiliations:** Department of Materials Science, Fudan University Shanghai 200433 China gaosp@fudan.edu.cn; Min H. Kao Department of Electrical Engineering and Computer Science, University of Tennessee Knoxville Tennessee 37996 USA ggu1@utk.edu

## Abstract

The present work exemplifies complementary perspectives offered by the band and bond pictures of solids, with an emphasis on the chemical intuition pertaining to the latter, especially in the presence of interfaces. The modern computational method of constructing a unique set of maximally localized Wannier functions from delocalized band states imparts new interpretations to the familiar concept of chemical bonds in the context of crystalline solids. By bridging the band and bond pictures using advanced computational tools, we reveal for the first time the unusual bond characters of a long-predicted fivefold coordinated structure of binary octet compounds A^*N*^B^8−*N*^ consisting of AA′ stacked planar AB honeycombs. While the isolated monolayer retains the familiar p_*z*_–π bonding in a honeycomb framework as in graphene and hexagonal boron nitride, the bulk foregoes in-plane π bonding and embraces out-of-plane ⋯A–B–A–B⋯ chain bonding *via* overlapping p_*z*_ orbitals. Not only does the chemical intuition gained by invoking the bond picture clarify the chemical nature of the fivefold coordination, but it also facilely explains a salient discrepancy in theoretical predictions in otherwise sound ample experimental evidence in the form of epitaxial thin films, paving the way towards rational synthesis of such thin films for optoelectronic applications. On the other hand, we show that the conduction band minimum, important in determining the electrical and optical properties, is a distinctly extended state that can only be properly described within the band picture.

## Abstract 2

A refreshed view of chemical bonds in solids unveils unusual bonding in fivefold coordinated bulk crystals of octet compounds: out-of-plane cation–anion chain bonding *via* overlapping p_*z*_ orbitals replaces in-plane π bonding of the isolated monolayer.

## Graphical abstract

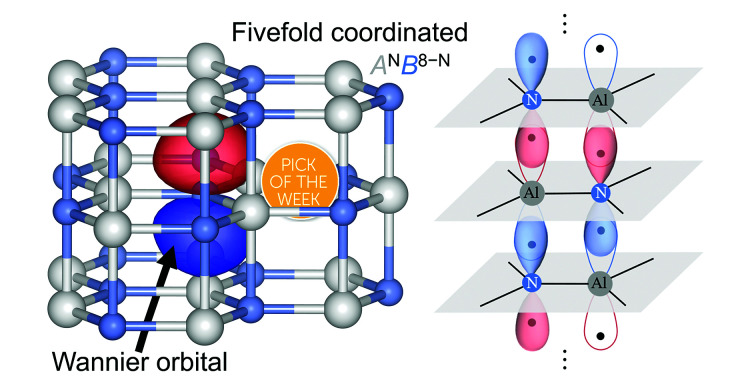

## Introduction

**Fig. 1:**
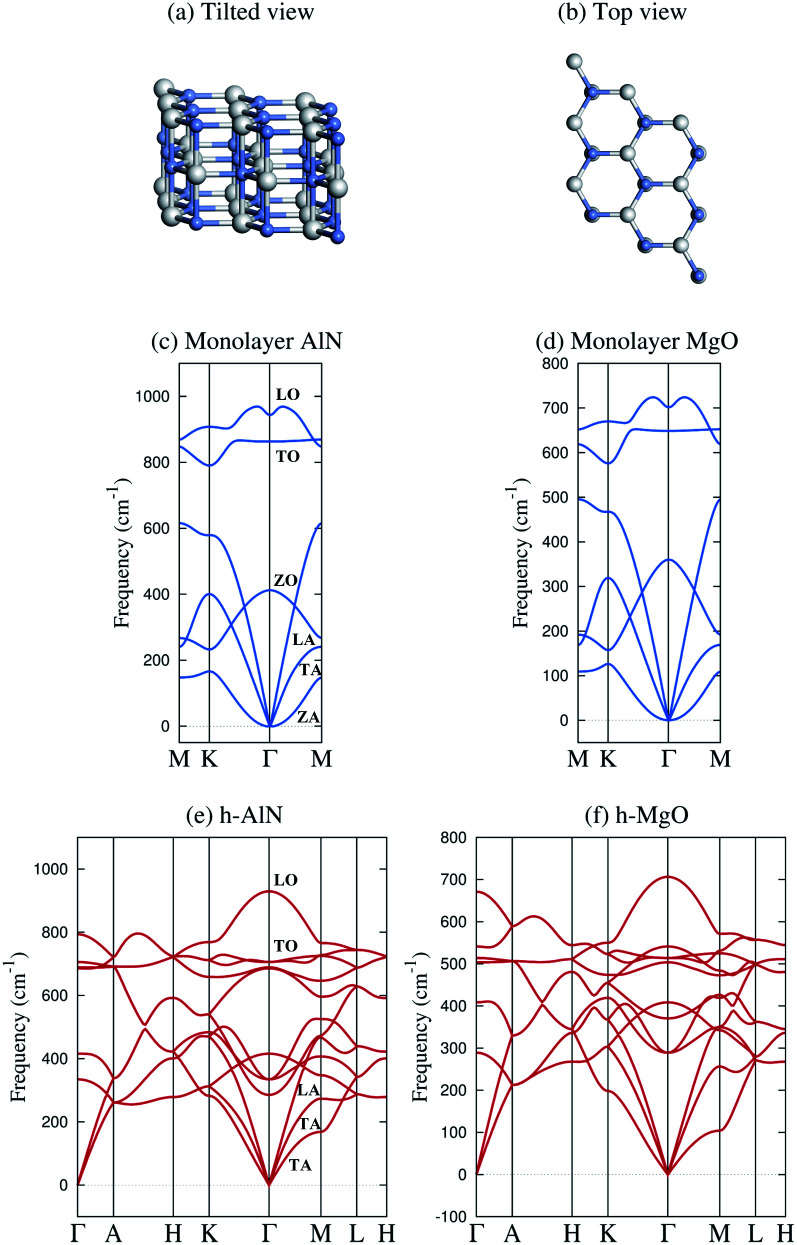
Schematic illustration and representative phonon dispersions of the h-MgO structure. (a) Tilted and (b) top views. (c and d) Monolayer h-AlN and h-MgO phonon dispersions. (e and f) Bulk h-AlN and h-MgO phonon dispersions. A striking feature of the bulk dispersions is the lack of resemblance to corresponding monolayers and the absence of interlayer modes, indicating strong bonding in the *c* direction. The monolayer dispersions (and those in Fig. S1†) are consistent with studies on the monolayers^24,25^ and the bulk h-AlN result agrees with that reported by Bacaksiz *et al.*,^3^ except that we take into account the LO-TO splitting at the *Γ* point, a typical feature of binary compound crystals.

The band and bond pictures of solids offer complementary perspectives, with the latter conducive to chemical intuition especially in the presence of interfaces and defects.^1,2^ Chemical bond characters of a material, not apparent from the extended energy and momentum eigenstates in the band picture, can provide insight into the structure, properties, and synthesis of the material, as we shall show here for a fivefold coordinated phase of binary octet compounds A^*N*^B^8−*N*^ consisting of AA′ stacked planar AB honeycombs^3–5^ (Fig. 1a and b).

Under ambient conditions, group IV–IV, III–V, and many II–VI binary octets exhibit fourfold coordinated polymorphs including zinc blende and wurtzite, as well as numerous stacking variations,^6–8^ whereas higher ionicity compounds are stable in structures of higher coordination numbers (*e.g.* MgO in the NaCl structure). Boron nitride is unique due to the sp^2^-coordinated, AA′-stacked layered polymorph h-BN; it is so stable that it had been unclear whether h-BN or the zinc blende phase (z-BN) is the thermodynamically stable phase until z-BN was generally accepted as such.^9^

The fivefold coordinated structure is an intermediate predicted for some A^*N*^B^8−*N*^ between phases characterized by coordination numbers *N*_c_ = 4 and 6, along the paths of structural transitions exhibiting a general trend of increasing *N*_c_ with increasing ionicity upon successive compression.^4,5,10–17^ This structure has the same symmetry (*P*6_3_/*mmc*) as h-BN and is referred to as the h-MgO structure (just as NaCl for the rocksalt structure, the compound not necessarily MgO) since it was first predicted for MgO^4^ or as the 5-5 structure for the mutual fivefold coordination,^5,16–19^ or simply as HX standing for hexagonal.^11^ As the present work focuses on bonding, we emphasize that the h-MgO structure is distinct from that of h-BN, consisting of threefold coordinated layers held together by a weak van der Waals (vdW) interlayer interaction. A compilation of the literature reveals the overall trend as well as the positions of the h-BN (*N*_c_ = 3) and h-MgO (*N*_c_ = 5) structures along the paths (see the ESI† for details).

Previous theoretical studies addressed crystal and electronic structures,^3–5^ whereas the characters of chemical bonds, especially the unusual bonds between the adjacent (0001) planes, are largely left unexplored, with fivefold coordination defined loosely as having a near-unity in-plane to vertical (*c*-direction) bond length ratio.^4,18^ For octet rule obeying A^*N*^B^8−*N*^, it is intuitive to attribute the threefold symmetry around *c* to σ bonding of sp^2^ orbitals in the basal plane. What is, then, the nature of the “interlayer” bonding, and what happens to the p_*z*_ orbitals? Here, we loosely use the term “interlayer” to mean inter-basal-plane for simplicity, by no means deemphasizing the “nonlayeredness” of the h-MgO structure. Bacaksiz *et al.*^3^ suggested the dominance of ionic interactions in the interlayer bonding of h-AlN. Limpijumnong *et al.* found evidence for additional chemical bond formation upon transitions from w-MgO to h-MgO and then to rocksalt MgO.^4^ They attributed the formation of additional bonds (interlayer bonds in the case of w-MgO → h-MgO transition) to increased ionicity, although pointing out that noticeable covalent bonding was apparent. Nevertheless, the character of the interlayer bonds in crystals of the h-MgO structure has not been revealed.

Experimental evidence has been ample but only in the form of ultrathin films or nanostructures.^20–23^ A salient common discrepancy in experimental demonstrations of the h-MgO structure is the absence of structural evolution with increasing thickness predicted by theory. In all cases where ultrathin A^*N*^B^8−*N*^ films are deposited on noble metal surfaces, only up to two bottom monolayers have been experimentally confirmed to exhibit a planar structure with the calculated lattice parameters, whereas the measured lattice parameters quickly approach the wurtzite values as more layers are deposited. Buckling is observable in some cases. The deviation from the h-MgO structure towards the wurtzite phase occurs well below the thickness threshold up to which the h-MgO structure is theoretically shown to be thermodynamically stable.^26^ The discrepancy persists even when substrates are considered in theoretical calculations.

Binary octet compounds along with their alloys span over a wide spectrum of band gaps that suits a myriad of electronic and photonic applications.^27–29^ Exhibiting differing physical properties,^6^ polymorphs of a compound are desired for different applications.^30–32^ While fourfold coordinated zinc blende and wurtzite phases as well as numerous stacking variations are used in current technologies, the search for novel polymorphs is a lasting endeavor.^14,32,33^ Compared with the wurtzite phase, the fivefold coordinated structure possesses higher symmetry that results in technologically favorable physical properties (*e.g.*, absence of spontaneous polarization) for certain applications.

In this work, we focus on h-AlN and h-MgO, each a metastable phase under ambient conditions of a compound of pre-transition elements, to elucidate bonding dictated by s and p electrons. Using *ab initio* calculations (see the ESI† for Computational details), we first show that their phonon dispersions lack the signatures of vdW layered crystals, clarifying the connotation of fivefold coordination beyond the in-plane to vertical nearest neighbor distance ratio criterion used in the literature.^4,5^ Simulated core level transition spectra reveal σ and π bonding, typical of threefold sp^2^ coordination, in isolated monolayer (1L) h-AlN and h-MgO, as in h-BN, but the signatures of this bonding scheme are missing in the bulk crystals. Not only do the bulk band structures bear less resemblance to the corresponding 1L sheets than to the wurtzite counterparts, but also the energy dispersions in the *c* direction are even stronger in bulk h-AlN and h-MgO than in the wurtzites, manifesting a stronger interlayer interaction in the fivefold coordinated phase than the fourfold coordinated phase. Maximally localized Wannier functions (MLWFs)^1^ of the bulk crystals confirm the absence of p_*z*_–π bonding while visualizing sp^2^–σ bonding similar to that in h-BN. In resolving this “missing π” mystery, we have revealed the peculiar bonding of this structure by bridging the band and bond pictures using angular momentum-resolved projection of band states onto atom centered local orbitals. The unusual bonding picture sheds new light on the occurrence of this crystal structure beyond the common argument of ionicity and due to the crucial role played by interfacial bonding in epitaxy and also on the synthesis of these materials as epitaxial thin films for utilization in semiconductor devices.

## Results and discussion

Fig. 1 displays phonon dispersions for bulk and monolayer h-AlN and h-MgO, representative of the h-MgO type of structure. The absence of imaginary frequencies indicates dynamical stability. In contrast, the instability of bulk h-InN, h-GaN, and h-ZnO is revealed by imaginary frequencies, despite stable monolayers (Fig. S1†). In addition to h-AlN and h-MgO that this work focuses on, we have discovered h-CaO, h-SrO, and h-CdO that are stable from monolayer to the bulk limit. While the search for the factors determining the stability of the h-MgO structure polymorphs is beyond the scope of this work, we point out that a stable monolayer is not a prerequisite for a stable bulk, as exemplified by h-BaO, suggesting that interlayer interactions play an important role. There are compounds, such as ZnS, that are unstable in the h-MgO structure as either monolayer or bulk under ambient conditions. Table 1 lists the free-standing 1L and bulk dynamical stabilities of representatives of those dynamically stable in one of the two limits, along with lattice parameters calculated in this work; values for h-BN are also provided for reference.

**Table 1:** Calculated lattice parameters^a^ of the h-MgO and wurtzite phases of the selected octet compounds, in comparison with representative prior calculations and experiments (if available)

	*a*_h_	*c*_h_	*a*_1L_	*a*_w_	*c*_w_	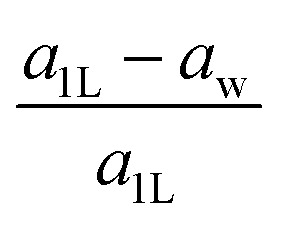
AlN^b^^,^^c^ (this work)	3.294	4.130	3.123	3.108	4.976	0.0048
Prior calculation^3^^,^^d^	3.30	4.15	3.13	3.11	5.01	0.0064
Experiment			3.14 (ref. 21)	3.112 (ref. 6)	4.982 (ref. 6)	0.0096
MgO^b^^,^^c^ (this work)	3.486	4.199	3.294	3.296	5.023	−0.0009
Prior calculation^20^	3.49		3.26			
Experiment^20^			3.25			
CaO^b^^,^^c^	3.961	4.767	3.762	—	—	—
SrO^b^^,^^c^	4.251	5.144	4.039	—	—	—
BaO^b^	4.534	5.592	4.304	—	—	—
CdO^b^^,^^c^	3.870	4.790	3.666	3.649	5.728	0.0047
ZnO^c^ (this work)	3.427	4.532	3.275	3.251	5.248	0.0071
Prior calculation^19^	3.48	4.54				
Experiment			3.29 (ref. 23)	3.252 (ref. 6)	5.213 (ref. 6)	0.012
BN (this work)	2.505	6.612	2.508	2.541	4.202	

a Here *a* and *c* stand for basal-plane and [0001] direction lattice parameters (in Å), respectively, while the subscripts h, 1L, and w signify the bulk h-MgO type of structure (or h-BN for BN), isolated monolayer, and wurtzite structure. Calculated *a*_1L_ values are for free-standing sheets, whereas experimental values are influenced by substrates, as discussed in the ESI.

b Bulk h-MgO type structure dynamically stable.

c Free-standing monolayer dynamically stable.

d Prior results including *a*_1L_ listed here for completeness; the bulk h-MgO structure phase and wurtzite values are also consistent with ref. 16.

In stark contrast to vdW layered crystals, the phonon dispersions of these hexagonal crystals lack the hallmarks of weak interlayer interactions. The out-of-plane ZA vibration modes of all stable monolayers as shown in Fig. 1 and S1† exhibit *ω* ∝ *q*^2^ frequency–wavevector dependence near the *Γ* point, as do all 1L 2D crystals, such as graphene^34^ and 1L h-BN,^35^ due to rapid decay of transversal forces.^25,34,35^ Layered crystals preserve the ZA mode, in which vibrations in the two layers of the unit cell are in phase, with the *ω* ∝ *q*^2^ dispersion nearly the same as that of the constituent monolayer.^34^ The mode in which adjacent layers of a layered crystal are out of phase and split slightly at *Γ* from the ZA mode due to the finite but weak interlayer interactions is referred to as the ZO′ mode by Mounet and Marzari.^34^ These two interlayer modes are widely known as the ripple or bending modes^36^ and are exemplified by graphite^34^ and bulk h-BN.^35^ None of the acoustic phonon branches of any of the bulk crystals as shown in Fig. 1 and S1† exhibit a superlinear dispersion or a soft ZO′ mode near *Γ*. Instead, each exhibits three acoustic branches with linear dispersion at *Γ* for in-plane wavevectors, just as conventional 3D crystals do.

The lack of soft, low-frequency interlayer modes propagating in the vertical direction is also very revealing. Layered crystals exhibit breathing (LO′ and LA)^34^ and shear (TO′ and TA)^34^ modes along *ΓA*.^34–37^ None of these low-frequency modes are found in any of the bulk crystals as shown in Fig. 1 and S1.† Instead, the *ΓA* dispersions resemble 3D crystals and exhibit group velocities comparable to the in-plane propagating modes, once again indicating strong “interlayer” interactions.

**Fig. 2:**
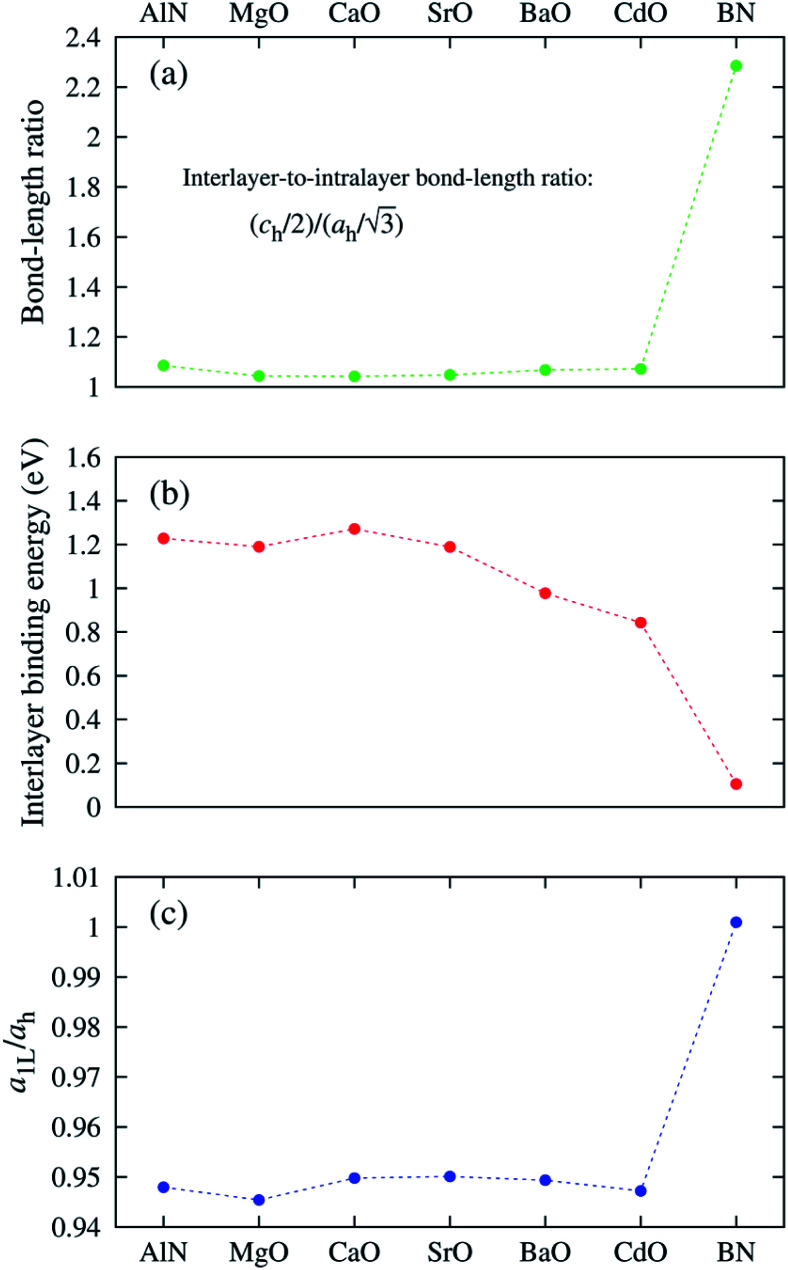
Structural features of the h-MgO structure in contrast to those of h-BN. (a) Ratios of the interlayer distance (*c*_h_/2) to intralayer bond length 
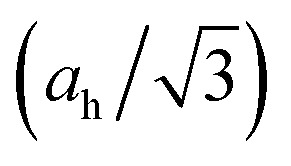
. (b) Interlayer binding energies, defined as monolayer-to-bulk total energy difference per cation–anion pair. (c) Monolayer-to-bulk lattice parameter ratios (*a*_1L_/*a*_h_).

The “nonlayeredness” of the h-MgO structure is also indicated by lattice parameter (Table 1) ratios and binding energies in the *c* direction (Fig. 2). In sharp contrast to the layered crystal h-BN, all these h-MgO type crystals exhibit interlayer-to-intralayer bond length ratios near unity, indicating fivefold coordination. Concurrently, the interlayer binding energies are one order of magnitude higher than that of h-BN, which is representative of the vdW interaction. Furthermore, as to be discussed later, the formation of interlayer bonds alters the in-plane bond lengths from the corresponding monolayer values, as revealed by the monolayer-to-bulk in-plane lattice parameter ratio in Fig. 2c.

**Fig. 3:**
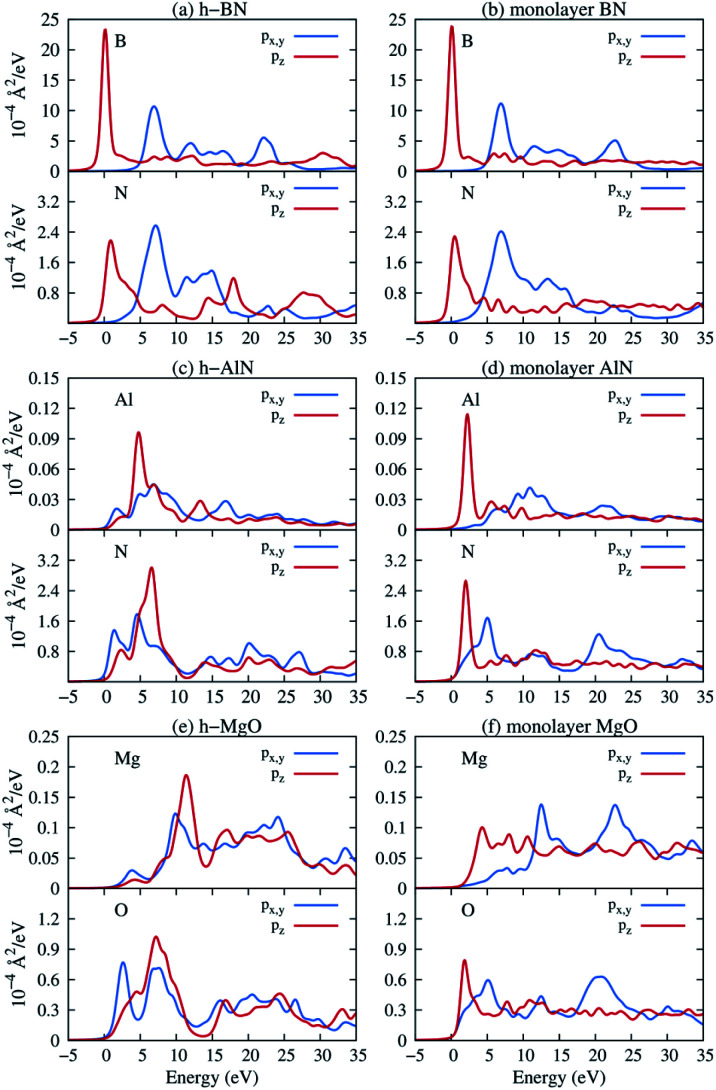
Near K-edge fine structure core-level spectra of 1L and bulk hexagonal crystals: (a and b) bulk and 1L h-BN, (c and d) h-AlN, and (e and f) h-MgO. Transitions from the 1s core orbitals to p_*x*,*y*_- and p_*z*_-projected unoccupied states are plotted separately. Energy is referenced to the lowest transition energy.

Next, we consider one particular experimental implication of the interlayer bonding. Core-level spectroscopy^38^ such as electron energy-loss spectroscopy (EELS) or, equivalently, X-ray absorption spectroscopy (XAS) has been widely used to analyse chemical structures of solids. Therefore, calculated spectra will guide the experimental verification of the synthesis of these materials. In EELS, the energy loss near-edge structure (ELNES), by replicating the density of unoccupied states,^39,40^ reveals the electronic structure of unoccupied states thus providing reliable information on bonding and coordination.^41^ As discussed in the ESI,† under the dipole approximation,^42,43^ angle-resolved near-edge spectra are closely related to densities of states projected onto p_*z*_- and p_*x*,*y*_-like symmetries, whereby transition peaks are identified as originating from π* (p_*z*_) and σ* (p_*x*,*y*_) states.^43–47^ For 2D and layered materials such as graphene, graphite, and h-BN, both angle-resolved ELNES and polarization-resolved XANES have experimentally revealed π* and σ* transitions, as predicted by density functional theory (DFT) calculations with core hole effects properly considered.^44,47,48^Fig. 3 shows calculated core-level transition spectra of h-AlN and h-MgO in comparison with those of h-BN, in which the p_*z*_ edges are lower in energy than the p_*x*,*y*_ ones, consistent with the familiar energy sequence^1,38,49^ that π* states are lower than σ*. Both the p_*z*_- and p_*x*,*y*_-like spectra of bulk h-BN are very similar to their respective 1L counterparts, due to the weak vdW interlayer interaction. While 1L h-AlN and h-MgO share this energy sequence (p_*x*,*y*_ higher than p_*z*_), manifesting σ and π bond formation from sp^2^ and p_*z*_ orbitals, respectively, the bulks exhibit a reversed sequence, which, along with the longer in-plane cation–anion bonds in bulks than in monolayers (Fig. 2c), indicates that the bonding in the nonlayered bulks must be altered from the familiar model of σ and π bonds for the corresponding monolayers.

The above strong indicators of interlayer bonding, along with the anomaly in core-level transition spectra, warrant an investigation into the chemical bond characters of the h-MgO structure. The in-plane 120° angle suggests sp^2^ coordination, which is verified by simulated transition spectra of 1L h-AlN and h-MgO. If the interlayer interaction in the bulk materials is essentially ionic, so should be the in-plane bonding since the bond length ratio is near unity. If, however, the 120° coordination originates from sp^2^–σ bonding rather than just due to inherited symmetry from the constituent monolayer, what bonds are formed by p_*z*_ orbitals? We answer these questions in the following sections by bridging the bond and band pictures of solids.

Fig. S2† displays the band structures of the monolayer and bulk of the h-MgO phase followed by those of the wurtzite phase, in each column, of compounds BN, AlN and MgO in the rows for comparison. While 1L h-BN, h-AlN, and h-MgO all exhibit similar valence band structures originating from threefold coordinated σ and π bonding, bulk h-AlN and h-MgO bear less resemblance in band structures to their monolayers, surprisingly, than to their wurtzite counterparts. In contrast to the layered material h-BN,^50,51^ bulk h-AlN and h-MgO exhibit strong dispersion along *ΓA*, *HK*, and *ML* in the *c* direction. Moreover, both bulk h-AlN and h-MgO exhibit wider valence band energy spans along each of these lines than their respective wurtzite counterparts, indicating a stronger interlayer interaction for the fivefold coordinated polymorph than for the fourfold.

**Fig. 4:**
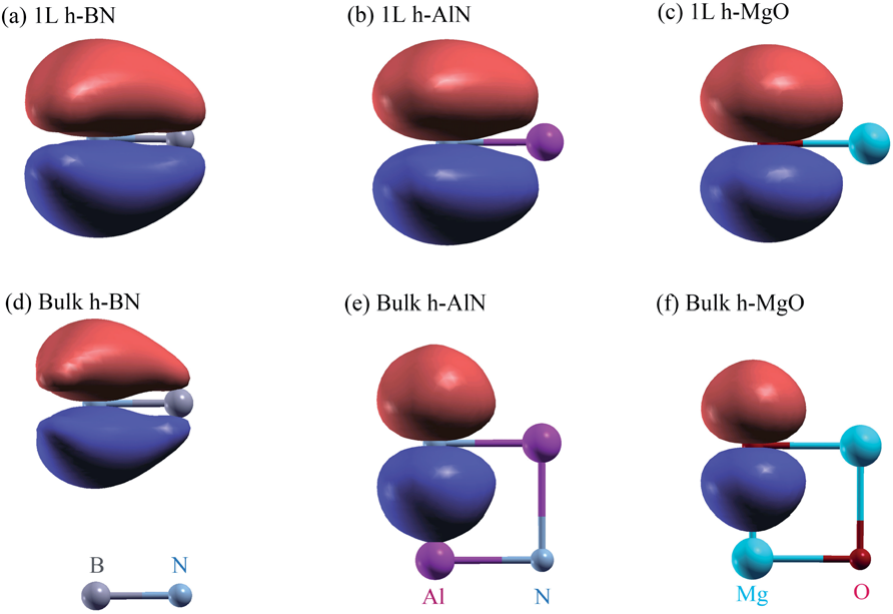
Isosurface plots of p_*z*_ Wannier orbitals in (a–c) 1L and (d–f) bulk h-BN, h-AlN, and h-MgO, each showing the side view of one primitive unit cell, with colored spheres representing the atoms labeled at the bottom of the figure. For better visualization in structural context, the TOC graphic displays one N-centered Wannier isosurface in the 3D model for each of 1L and bulk h-AlN. All isosurfaces are at the same isovalue, ± 0.15 (e Å^−3^)^1/2^, with signs indicated by red (+) and blue (−) colors.

**Fig. 5:**
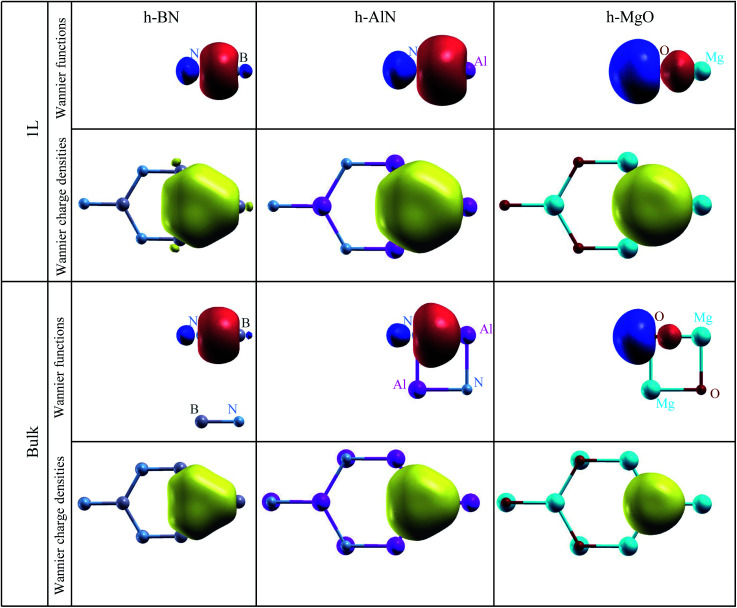
Isosurface plots of σ bond Wannier functions and Wannier electron charge densities of 1L and bulk h-BN, h-AlN, and h-MgO. All Wannier function isosurfaces are at the same isovalue, ±0.15 (e Å^−3^)^1/2^, with signs indicated by red (+) and blue (−) colors, and the charge density isovalue is 0.0675 e Å^−3^ (= 0.15^2^ × 3 e Å^−3^, exactly corresponding to the Wannier function isovalue, as total charge density of three σ bond Wannier orbitals is plotted). Colored spheres representing the atoms are labeled for clarity. These σ bond MLWFs are simultaneously generated with the p_*z*_ Wannier orbitals as shown in Fig. 4 but without symmetry (reflection with regard to the atomic plane) adaption.

To visualize the nature of chemical bonding with MLWFs,^1^Fig. 4 depicts symmetry-adapted,^52^ anion-centered p_*z*_ Wannier orbitals of h-BN, h-AlN, and h-MgO, and Fig. 5 shows the in-plane bond-centered sp^2^ MLWFs simultaneously generated without symmetry adaption. For h-BN, the in-plane MLWFs indicate σ bonding *via* sp^2^ hybridization, consistent with that reported by Halo *et al.*^53^ and reminiscent of graphene.^1^ The sp^2^ Wannier orbitals of the 1L and the bulk look similar to each other and so do the p_*z*_ orbitals, visualizing a familiar picture,^9^ where the σ bond forms *via* sp^2^ electron transfer from B to N whereas N atoms share p_*z*_ electrons with B to form π bonds, although the σ bond appears less covalent in bulk than in 1L h-BN. As ionicity increases from h-BN to h-AlN and then to h-MgO, the σ bond center shifts further towards the anion (Fig. 5), but the tendency for the anion to donate p_*z*_ electrons can still be seen for the isolated monolayers albeit lesser in 1L h-MgO (Fig. 4). While the familiar picture of σ and π bonding persists for the monolayers, bulk h-AlN and h-MgO display a drastically different picture: the p_*z*_ Wannier orbitals are almost rotationally symmetric, with the 0.15 (e Å^−3^)^1/2^ isosurfaces not protruding towards the cations but reaching the adjacent layers, indicating the absence of π bonding and manifesting interlayer electron orbital overlaps. The localized Wannier orbitals indicate in-plane σ bonding in the bulk h-MgO structures and suggest interlayer p_*z*_ electron sharing.

**Fig. 6:**
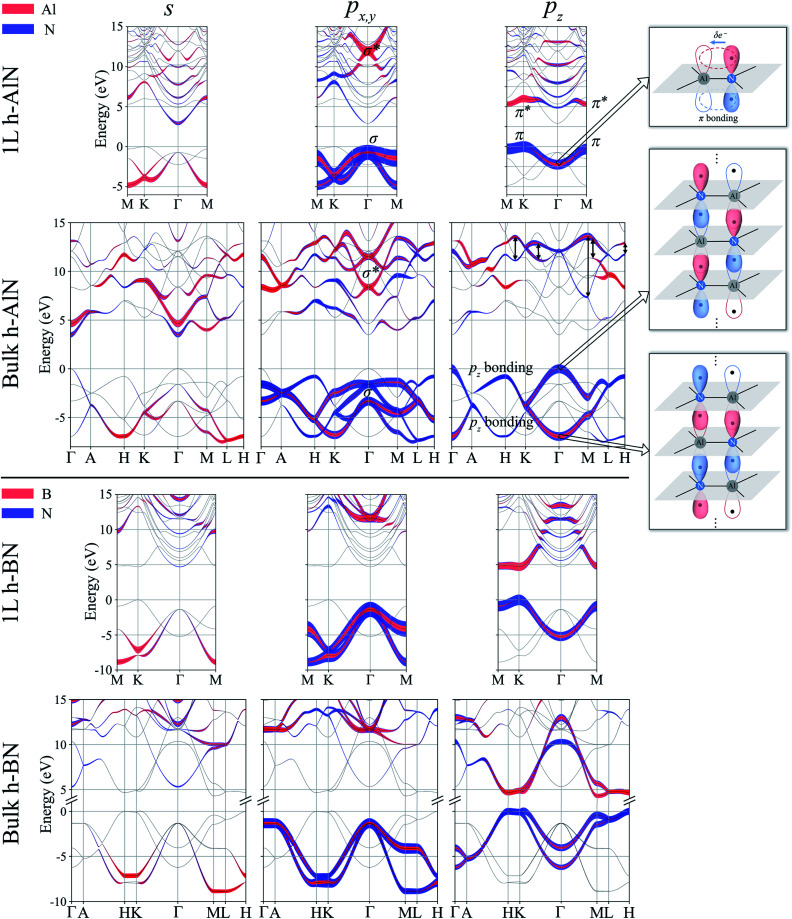
Fat band plots showing projected weights onto cation- and anion-centered s, p_*x*,*y*_, and p_*z*_ orbitals for 1L and bulk h-AlN (upper panel), compared with those of h-BN (lower panel). For h-AlN, band states with predominant σ, σ* and π, π* characters are labeled in the p_*x*,*y*_ and p_*z*_ plots, respectively. Going from 1L to bulk, the π band splits into two p_*z*_ bonding bands, and the π* band can be largely considered to split into two p_*z*_ antibonding bands as indicated by the double headed arrows; the p_*z*_ antibonding bands exhibit considerable mixing with σ* and free-electron-like bands. Bonding schemes derived from the projection are visualized on the right, where the σ bonds are depicted as thick black lines and the p_*z*_ orbitals as balloons with colors (red and blue) signifying orbital wavefunction signs, solid filling indicating filled orbitals, and each dot visualizing an electron.

Projecting extended Bloch states, especially those near band extrema, onto atomic orbitals is another way to reveal chemical bonding characters. Fig. S3† displays densities of states projected onto cation- and anion-centered local orbitals with s, p, and d characters (*i.e.* with angular momentum quantum numbers *l* = 0, 1, and 2) for h-BN and h-AlN in both 1L and bulk forms, alongside corresponding fat band plots. For both monolayers, the lower valence bands exhibit predominant s characters with minute p contributions, and the upper valence bands with a degenerate maximum at *Γ* are dominated by p states but mixed with s characters. To resolve the “missing π” mystery of bulk h-MgO structures, Fig. 6 presents fat band plots with p states further resolved into p_*x*,*y*_ and p_*z*_ states for h-AlN, compared with h-BN. As expected, for both 1L h-BN and h-AlN, the valence bands that peak at K stem from p_*z*_ states while those with maxima at *Γ* originate from p_*x*,*y*_, manifesting sp^2^ bonding. Upon the formation of bulk h-BN by stacking the monolayers, each valence band splits into two with a relatively narrow splitting. Each pair of split bands essentially exhibits the same sign of curvature, and all valence bands show minimal dispersion in the vertical direction (c*, along *ΓA*, *KH*, and *ML*). The in-plane splitting and c* direction dispersion are consistent with the weak interlayer interaction of h-BN.

For bulk h-AlN, with regard to the monolayer, the p_*z*_ valence band splits into two bands with opposite signs of curvature and a wide splitting of ∼7 eV at *Γ*, indicating strong interactions between p_*z*_ orbitals in neighboring atomic planes. The split p_*x*,*y*_ bands, on the other hand, exhibit the same sign of curvature (also the same as in 1L) and a smaller splitting of ∼2 eV at *Γ*, naturally following the smaller overlaps between p_*x*,*y*_ orbitals of neighboring layers. From these observations, emerge the bonding scheme of the fivefold coordinated h-MgO type structures of octet compounds A^*N*^B^8−*N*^, graphically illustrated for bulk h-AlN in Fig. 6. With the σ bond frameworks of constituent atomic planes intact, each anion B partially donates p_*z*_ electrons to cations A in neighboring layers, forming ⋯A–B–A–B⋯ cation–anion chains in the c direction. For the lower-energy p_*z*_ bonding states, the p_*z*_ orbitals of neighboring cations and anions are in phase and therefore this band exhibits the same curvature as the π bonding band in 1L h-AlN. Conversely, the higher energy p_*z*_ bonding band has neighboring p_*z*_ orbitals out of phase and accordingly an opposite curvature. The apparent shift of the VBM from K (for 1L h-AlN) towards *Γ* (for bulk h-AlN) is actually the splitting into multiple bands upon stacking of multiple layers, eventually resulting in the two p_*z*_ bonding bands in the bulk limit. The absence of π bonding naturally explains longer in-plane bonds in the bulk materials than in the corresponding isolated monolayers (*a*_1L_ < *a*_h_ in Fig. 2c).

In comparison with h-BN, where the AA′ stacking is dictated by electrostatic forces but the interlayer distance is set by vdW forces,^54^ for the fivefold coordinated h-MgO structures with increased ionicity, it is naturally understood that the stronger electrostatic interaction not only dictates the stacking and registration between neighboring honeycomb layers, but also forces interlayer distances to be smaller than those determined solely by vdW forces. In a gedanken process to assemble the bulk h-MgO structure from isolated monolayers, the p_*z*_ orbitals of the anions will significantly overlap those of the cations in neighboring layers, rendering preferred interlayer p_*z*_ electron sharing over the usual intralayer sharing that results in π bonding in the monolayers (Fig. 6). Nevertheless, the transition from in-plane π bonding in h-BN to vertical p_*z*_ chain bonding in h-MgO structures of higher-ionicity A^*N*^B^8−*N*^ is evolutionary rather than abrupt. We have found that h-BeO is the intermediate in between, which warrants separate investigations; this work focuses on the fivefold coordinated h-MgO type polymorphs that exhibit distinctive vertical p_*z*_ chain bonding.

We now examine the conduction bands of h-AlN, starting with the global conduction band minima (CBMs) of both the monolayer and bulk at *Γ*, around which the bands are parabolic and largely isotropic, even between the in-plane and vertical directions of the bulk. Furthermore, the CBMs exhibit virtually no p character and very low densities of states compared with higher empty band extrema. Bacaksiz *et al.*^3^ refer to these states as free-electron-like surface states. Here, we mention in passing that these states are indeed reminiscent of the free-electron states in graphene, graphite, and h-BN,^55^ with discussions to follow after we examine the p_*z*_ and sp^2^ antibonding states at higher energies. The s, p_*x*,*y*_ and p_*z*_ projected weights (Fig. 6) reveal sp^2^ and p_*z*_ antibonding characters of those higher conduction bands. Going from 1L to bulk h-AlN, the 1L σ* state around 12 eV at *Γ*, contributed by s and p_*x*,*y*_ projected states, splits by ∼3 eV, whereas the 1L π* band largely splits into two p_*z*_ antibonding bands as indicated by double headed arrows in Fig. 6. Similar to the p_*z*_ bonding bands, the two widely split antibonding bands have opposite curvatures, attributable to a lower- and higher-energy configuration with in-phase and out-of-phase in-plane neighboring p_*z*_ orbitals, respectively (Fig. S4†). Not surprising, the antibonding bands span over large ranges of energy and exhibit significant mixing with one another and with the free-electron-like states.

By now we can bring a close to the “missing π” mystery in the near-edge transition spectra of the h-MgO type crystals. The π bonding is indeed missing, giving way to interlayer p_*z*_ chain bonding. Going from 1L to bulk, the p_*z*_ bonding band maximum remains higher in energy than the σ band maximum, with the former band splitting farther than the latter. Due to the strong non-vdW interlayer p_*z*_ orbital interaction, the p_*z*_ antibonding-bonding splitting is not sufficiently smaller than the σ*–σ splitting to guarantee that the p_*z*_ antibonding states is lower-energy than that of σ*, in contrast to the σ–π systems of graphite and h-BN. Significant mixing between the σ* bands and the bands with free-electron-characterized minima, to be further discussed below, then leads to p_*x*,*y*_ transition peaks that are lower in energy than the p_*z*_ transition peaks.

**Fig. 7:**
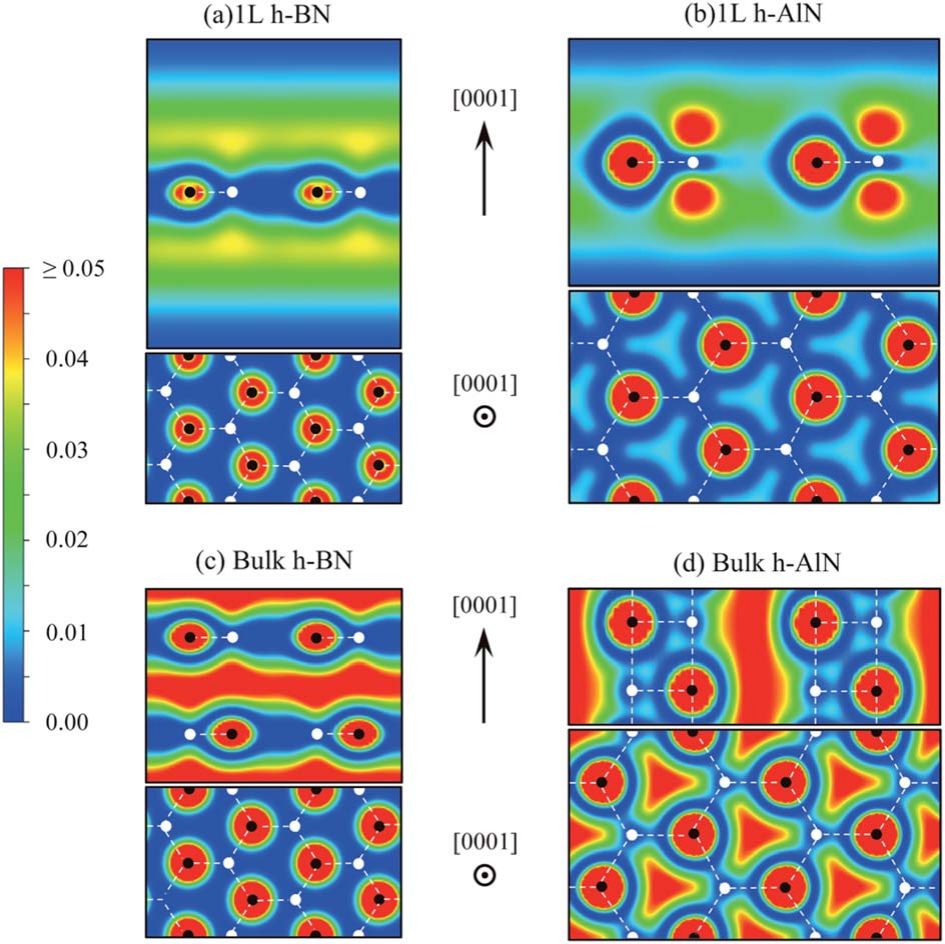
Charge density map (in e Å^−3^, color scale on the left) for free-electron-like states at *Γ* of (a) 1L h-BN and (c) bulk h-BN and for CBM states of (b) 1L h-AlN and (d) bulk h-AlN.

For further clarity, we now discuss the free-electron-like states, the counterparts of which have been known for decades in graphite/graphene^56,57^ and h-BN.^50,51^ These delocalized excited states, unaccounted for in linear combinations of localized 2s and 2p (for C and BN) orbitals due to basis incompleteness, can only be captured by using basis sets incorporating delocalized basis functions. For the graphene or h-BN monolayer (Fig. 7a), its electron density is concentrated around two planes on both sides of and parallel to the atomic plane. These highly delocalized states morph into interlayer and surface states in the case of graphite,^57^ but yield only interlayer states without forming surface states for bulk h-BN (Fig. 7c) because the wavefunctions are more “atomic” (more localized, less free electron like), resulting in the more Bloch-like interlayer states and no surface states extending into vacuum.^50^ It was realized early on that such unoccupied interlayer states were common in layered materials,^58,59^ and previous calculations of the honeycomb monolayers of a large collection of II–VI compounds yielded apparently parabolic empty bands.^24,25^ Our calculated h-AlN and h-MgO band structures now suggest that such parabolic, largely isotropic bands of the isolated monolayers and the corresponding bulks are a common feature also to the h-MgO family of planar materials, even without vdW gaps in the crystals.

The abovementioned trend of more “atomic” interlayer states with increasing ionicity from graphite to h-BN extends to h-AlN. Fig. 7b and d show the charge density distributions of the CBM states for h-AlN, in comparison with those of h-BN, which agree with previous calculations.^50,51^ The interlayer high-electron-density regions of bulk h-BN already show some lateral (*i.e.* in directions parallel to the basal plane) variation in electron density due to ionicity, compared with graphite.^57^ With further increased ionicity, the 1L h-AlN free-electron-like state is not planar. The decreased “interlayer” distance (*c*/2) of h-AlN (with regard to h-BN) further breaks the planarity of the free-electron-like states due to the increased influence of the crystal potential, as can be seen with a 1D model (in the *c* direction)^51^ used to reveal the physical nature of such states in h-BN. Fig. 7 indicates that it is no longer appropriate to refer to these states in h-AlN as interlayer states; rather, a descriptive term is “channel states”.

The free-electron-like states in bulk h-MgO type polymorphs of A^*N*^B^8−*N*^ play a critical role in determining the physical properties of these materials, as CBM states, together with the VBM states. The CBM–VBM colocation in the reciprocal space at *Γ* imparts direct band gaps, desirable for potential optoelectronic applications, to the bulk materials. This important role of the channel states is in sharp contrast to the interlayer states of graphite well above the lowest π* state near *K* and thus with virtually no implication on materials properties, and is also different from the interlayer states of h-BN that compete against the lowest π* states to claim the CBM.^51^

In the above sections we bring to light the unusual bonding scheme (Fig. 6) in h-MgO structures, in which sp^2^ coordinated, σ bonded planar A^*N*^B^8−*N*^ monolayers are strongly linked together in the *c* direction by out-of-plane p_*z*_ orbital interaction forming ⋯A–B–A–B⋯ cation–anion chains. Not only does the VBM shift to *Γ* from *K*, losing the hallmark of π bonding in honeycomb lattices, but it also exhibits smaller effective mass along *ΓA* than in the in-plane directions, indicating that the p_*z*_ electrons are more delocalized along *c* than laterally. High hole mobility along *c* is therefore expected. On the other hand, the electron transport dominated by the free-electron-like channel states around CBM is expected to be isotropic and exhibit high mobility. Along with the direct band gaps at *Γ*, the charge transport properties of these nonlayered materials promise potential applications in optoelectronic devices, especially in those device structures where the current flows along c. Furthermore, GW corrected calculations yield band gaps similar to those of their wurtzite counterparts (Fig. S2†), allowing for applications in the same spectral ranges.

The unusual chemical bonding is revealed by bridging the band and bond pictures, which describe the electrons in basis sets of extended Bloch orbitals (common eigenstates of energy and momentum) and of localized bonding and antibonding orbitals, respectively. The two representations are related by a unitary transformation in principle. This relation between the two views of a solid is analogous to that between the molecular orbital (energy eigenstate) and bonding (localized) views of a molecule.^1^ Indeed, MLWFs are crystal analogs of Foster-Boys (FB) localized orbitals of molecules, as discussed in ref. 1 with FB localization exemplified by references therein; in an excellent additional example,^60^ the FB orbitals transformed from delocalized molecular orbitals were shown to correspond well to σ and π bonds and lone pairs in nucleotide bases. For both solids and molecules, the bonding view is conducive to chemical intuition. While the chemical bonding argument is essential to the explanation as to why the crystal structure occurs in the first place, as exemplified by the present work, it also appears self-fulfilling since electrons inevitably concentrate along lines connecting the nearest-neighbor atoms,^61^ and may be considered unnecessary for perfect crystals.^2^

The understanding of bonding, however, once obtained for a perfect crystal, *e.g.*, from first-principles band structures as carried out here, is essential to real-world crystals with defects^2^ including surfaces and interfaces, providing us a fresh perspective to examine the challenges encountered by experimental syntheses of the h-MgO structured A^*N*^B^8−*N*^. A noticeable commonality of experimental demonstrations of the h-MgO structure^20–23^ is the absence of structure evolution with increasing thickness predicted by theory. Table 1 shows that the isolated monolayers exhibit lattice parameters *a*_1L_ which are appreciably smaller than the bulk value *a*_h_, consistent with the trend from monolayer towards bulk with increasing thickness shown in previous calculations.^3,20–23^ For ultrathin MgO grown on Ag(111), only *a*_1L_ = 3.25 Å agrees with the calculated value, whereas *a*_5L_ = 3.28 Å at a 5 ML thickness is much smaller than the calculated value 3.43 Å along the evolution path towards *a*_h_ = 3.49 Å.^20^ Similarly, ultrathin AlN on Ag(111) (ref. 21) agrees with the prediction of h-AlN by *a*_1L_ = (3.14 ± 0.06) Å, but *a*_4L_ ≈ 3.13 Å measured at a 4 ML thickness contradicts the expected increase towards the bulk value (*a*_h_ = 3.294 Å, this work; *a*_h_ = 3.30 Å and *a*_2L_ = 3.20 Å, ref. 3). Furthermore, ultrathin ZnO nanosheets on Ag(111) and Au(111) both fail to show the expected progressive increase in the in-plane lattice parameter with increasing layer count,^22,23^ although h-ZnO is known to be energetically stable below a threshold thickness.^26^

We notice the close coincidence *a*_1L_ ≈ *a*_w_, the latter being the basal-plane lattice parameter of the wurtzite phase, with a relative difference (*a*_1L_ − *a*_w_)/*a*_1L_ < ∼0.7% for each compound in Table 1, readily understandable within our bonding picture (Fig. 6): cation–anion bonds in the monolayer are shortened by π bonding with regard to the planar sp^2^–σ bond framework in the bulk without π bonding, and the basal plane projection of the canted sp^3^–σ bond is shorter than the bond itself. While the h-MgO structure is the energetically stable phase at few-ML thicknesses, the growth may proceed towards wurtzite even at few-ML thicknesses *via* a kinetic path associated with the coincidence *a*_1L_ ≈ *a*_w_, if a free-energy barrier exists between the two phases, which is already implied by the bonding scheme without detailed calculations.

In each of the abovementioned experimental cases, ultrathin A^*N*^B^8−*N*^ grows on a metal surface, where the epi-substrate interactions alter the bonding in the epi. The p_*z*_ orbitals of the monolayer h-MgO structured epi mix with substrate orbitals and thus deviate from the p_*z*_–π bonding of the freestanding monolayer. The interaction of cations A and anions B with the substrate must differ. Considering a similar case of h-BN on metal surfaces,^62–64^ we infer that the p_*z*_ orbitals of cations A are pulled towards the substrate, resulting in sp^3^ mixing. Reminiscent of the wurtzite structure with A facing the substrate, this asymmetry may trigger wurtzite growth (Fig. S5†), even without observable buckling of the bottom ML in first-principles modeling. Indeed, the calculations that consider the substrates yield “almost planar” MgO sheets on Ag(111)^20^ and “very small corrugation” in ZnO on Au(111) “with a preference for O-termination”.^23^ For few-ML thicknesses, the tendency towards wurtzite growth is suppressed by the higher total energy of the wurtzite phase associated with the top surface dangling bonds (compared with the h-MgO phase top surface where the outward pointing p_*z*_ orbital lobes can share electrons laterally, reminiscent of π bonding). Therefore, the bottom MLs appear almost planar. In other words, the distinction between the h-MgO structure and wurtzite phases does not apply to very-few-ML A^*N*^B^8−*N*^ ultrathin films on such substrates. Taking advantage of the coincidence *a*_1L_ ≈ *a*_w_, however, the wurtzite phase wins the competition as thickness increases, as manifested by the in-plane lattice parameter remaining at or reverting to ∼*a*_1L_ ≈ *a*_w_ rather than increasing as expected for the genuine h-MgO structure. The general transition from planar bottom monolayers to wurtzite-like structures and the individual cases are discussed in greater detail in the ESI,† where we also touch on possible defect effects, to which the measurement-prediction discrepancy was attributed in one case.^20^

Lastly, we mention that the fivefold-coordinated bonding in h-MgO structured A^*N*^B^8−*N*^ crystals is different from the geometrically similar sp^3^d–σ bonding in molecules such as PX_5_ (X = F, Cl), at least for the compounds of the third period cations and second period anions the present work focuses on, as negligible d characters are found in the valence bands (Fig. S3†).

## Conclusions

By bridging the band and bond pictures of solids using modern computational means, including MLWF construction from localized (*l*, *m*)-resolved orbital projection of *ab initio* obtained band states, we reveal the unusual bonding of the fivefold coordinated phase of octet compounds A^*N*^B^8−*N*^. While the isolated monolayer, planar due to energetic preference, retains the familiar p_*z*_–π bonding between A and B in a framework of sp^2^–σ bonds, the freestanding multilayer or bulk of h-MgO structured A^*N*^B^8−*N*^ foregoes in-plane π bonding and embraces ⋯A–B–A–B⋯ chain bonding between atomic layers. This fresh bonding perspective is conducive to chemical intuition in dealing with surface, interface, and defect related phenomena. A common discrepancy with theory in the experiments involving ultrathin A^*N*^B^8−*N*^ films can now be facilely explained by a simple substrate effect: in early-stage epitaxial growth, even a minute difference between the interactions of A and B with the substrate tends to break the symmetry of the h-MgO structure and kinetically steer growth towards the wurtzite phase, facilitated by its proximity in the basal-plane lattice parameter to the monolayer (*a*_1L_ ≈ *a*_w_). The insight into the limitations in previous experiments points to rational strategies for reliably synthesizing large-area thin films of h-MgO structured A^*N*^B^8−*N*^ for potential optoelectronic applications, expected from more favorable properties entailed by their higher symmetry over the common wurtzite phase, as well as similar band structures (direct gap, GW-corrected gap value, *etc.*) to their widely used wurtzite counterparts. The application-favorable direct band gaps of the bulk fivefold coordinated A^*N*^B^8−*N*^ crystals are in part due to the free electron-like “channel states”, which are more conveniently studied in the band picture that complements the bond picture.

## Conflicts of interest

There are no conflicts to declare.

## Supplementary Material

SC-011-D0SC00292E-s001
