# Supplementary material for: Peculiar bond characters of fivefold coordinated octet compound crystals
Source: Chem Sci. 2020 Mar 27;11(17):4340–50. doi: 10.1039/d0sc00292e (PMC8152722; doi:10.1039/d0sc00292e)
Supplement: SC-011-D0SC00292E-s001 [file SC-011-D0SC00292E-s001.pdf]

Electronic Supplementary Material (ESI) for Chemical Science.
This journal is © The Royal Society of Chemistry 2020

Supplementary Information

Peculiar bond characters of fivefold coordinated octet compound crystals

An-An Sun,[a] Shang-Peng Gao*[a] and Gong Gu*[b]

[a] Department of Materials Science, Fudan University, Shanghai 200433, China

[b] Min H. Kao Department of Electrical Engineering and Computer Science, University of Tennessee, Knoxville, Tennessee 37996, USA

*Emails: gaosp@fudan.edu.cn; ggu1@utk.edu

Table of contents

Supplementary figures 2

Computational details 6

Literature on structural transitions upon ionicity increase induced by compression 7

Angle-resolved core-level spectroscopy 7

Epitaxial h-MgO structured A^NB^{8-N} ultrathin films on substrates 8

References 11

Supplementary figures

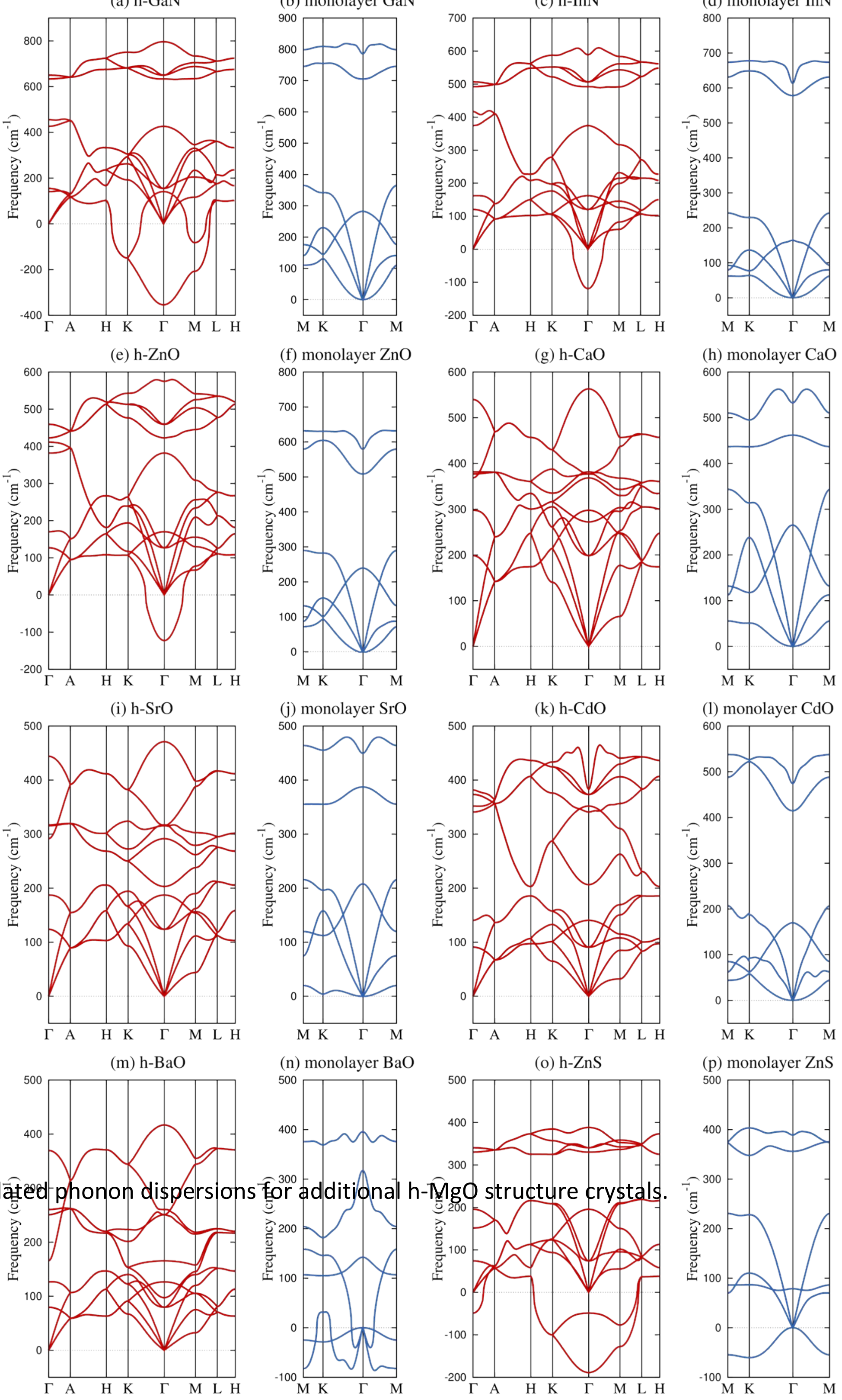

Fig. S1 Calculated phonon dispersions for additional h-MgO structure crystals.

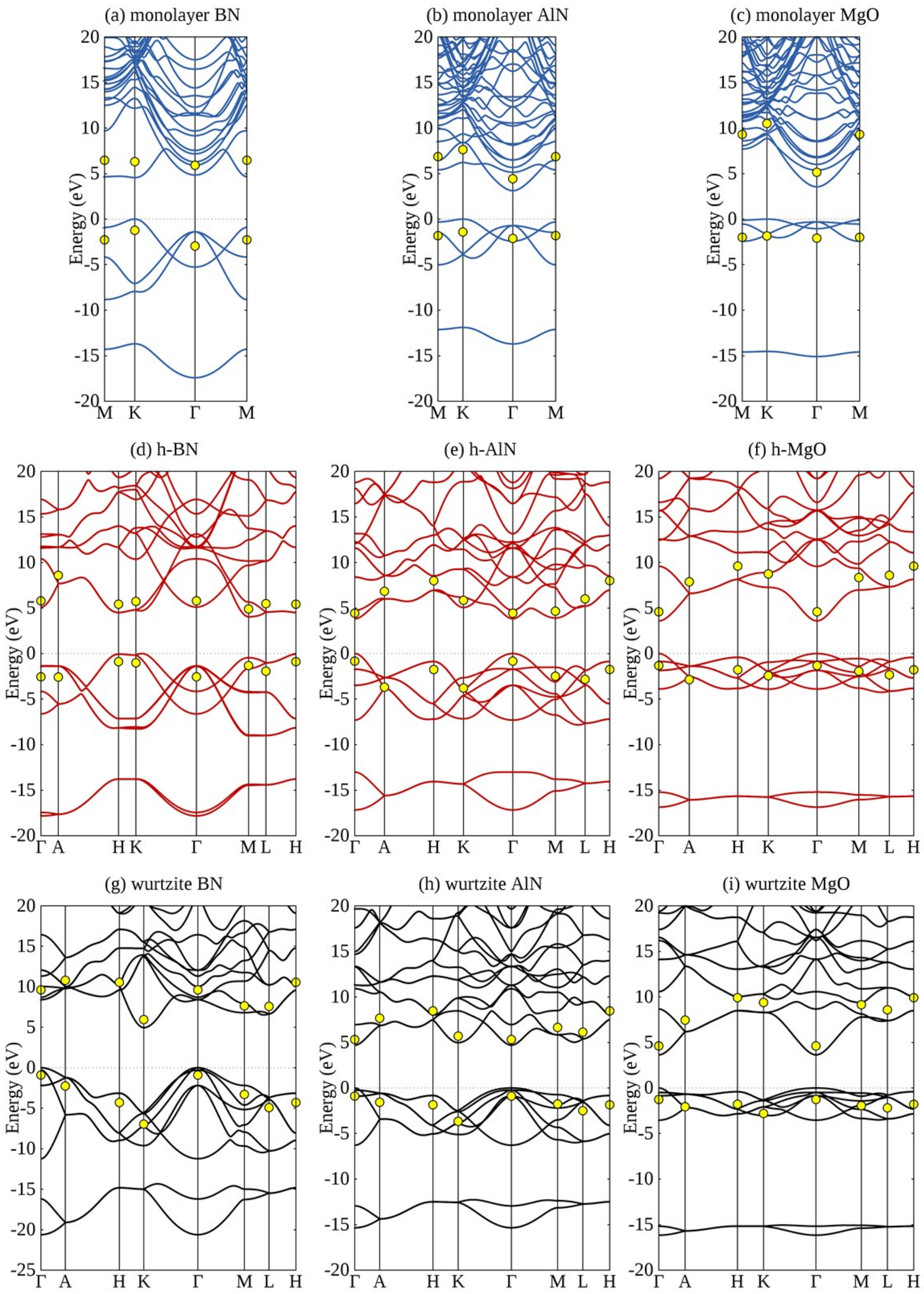

Fig. S2 Band structures of monolayer, bulk hexagonal planar, and wurtzite forms of BN, AlN, and MgO. Red, blue and black curves represent the LDA band dispersions of different structures. Yellow circles indicate quasiparticle energies at high-symmetry points for the highest valence and lowest conduction bands obtained from *GW* calculations. The LDA valence band maximum at 0 eV is indicated by a horizontal dotted line for clarity.

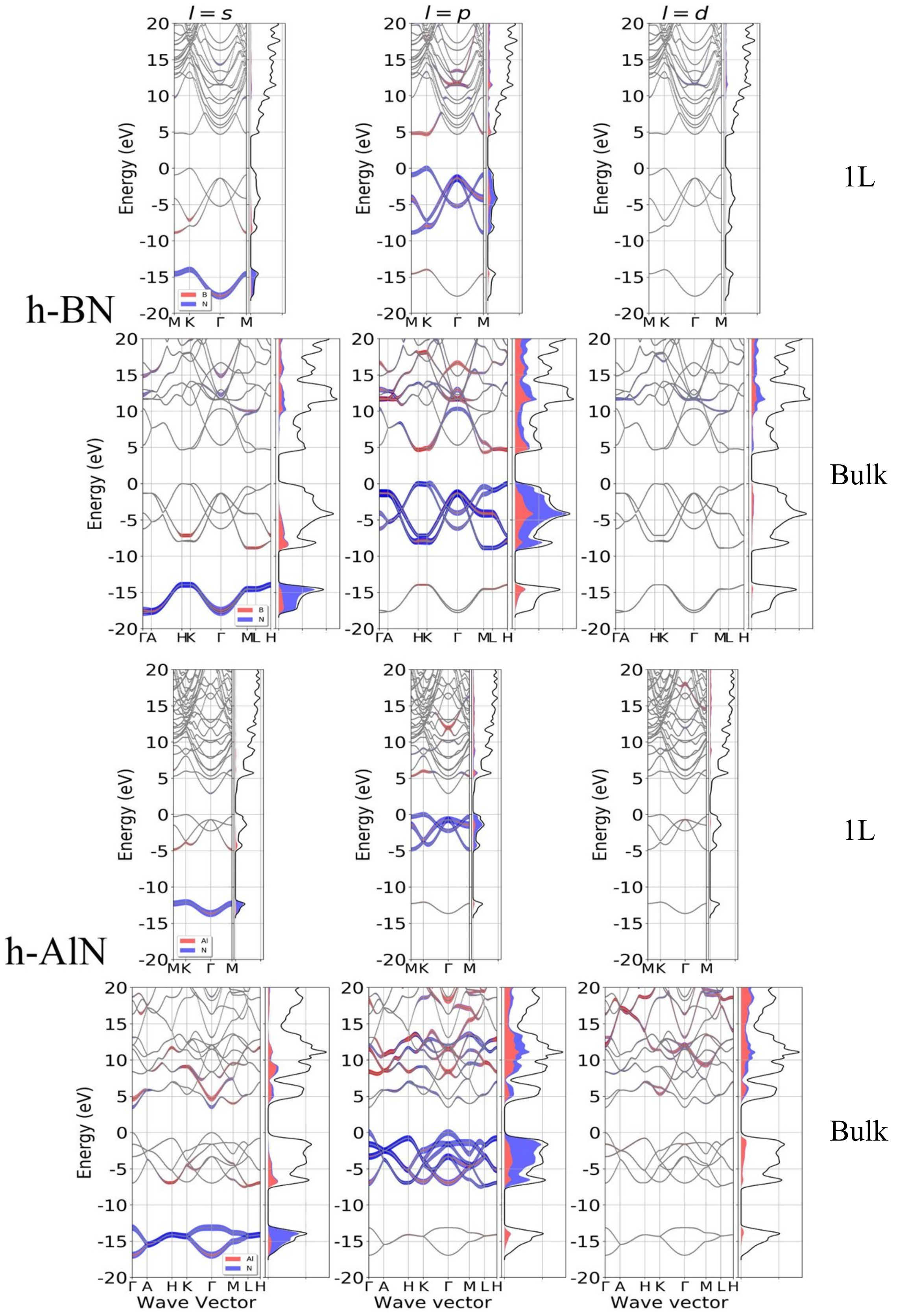

Fig. S3 Angular momentum-resolved fat band and PDOS of 1L and bulk h-BN, h-AlN.

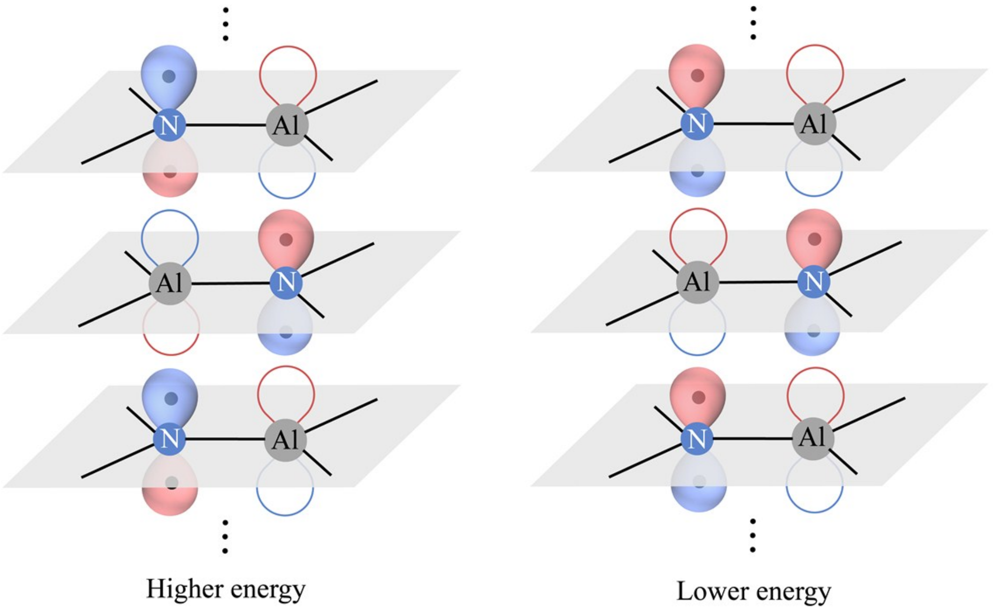

Fig. S4 Schematic illustrations of h-AlN antibonding states. The σ bonds are depicted as thick blacklines and the p_z orbitals as balloons with colors (red, blue) signifying orbital wavefunction signs, solid filling indicating filled orbitals, and each dot visualizing an electron.

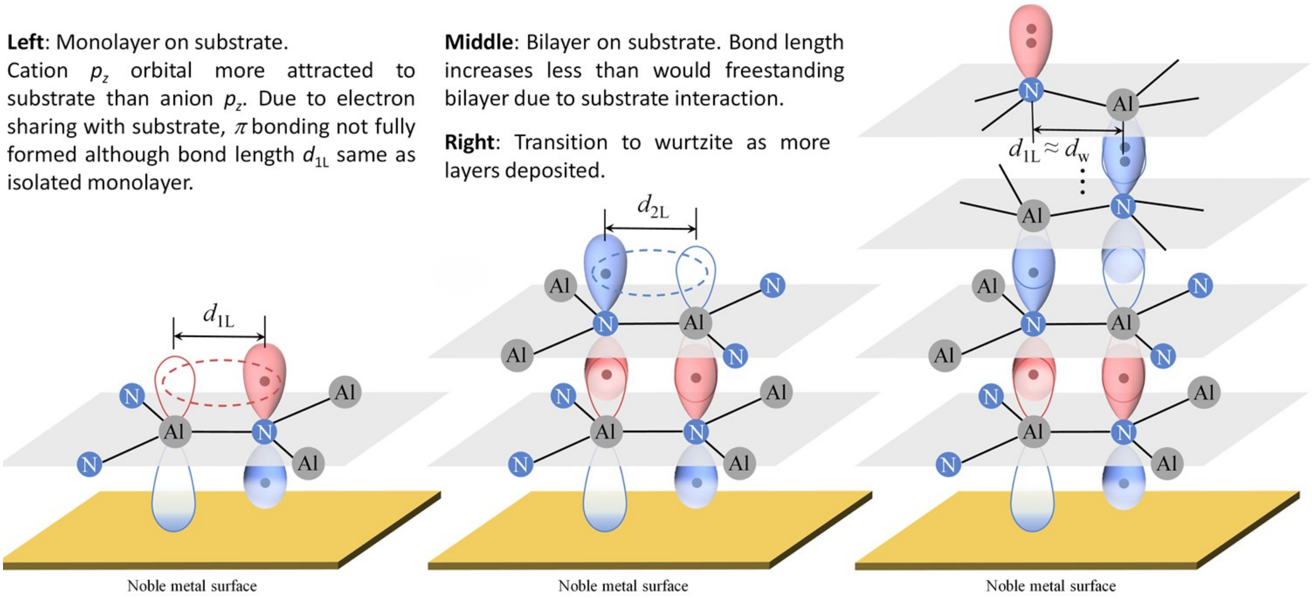

Fig. S5 Schematic illustration of evolution in bonding from monolayer, bilayer, to multilayer h-AlN on a noble metal surface.

Computational details

We perform structural, phonon, and electronic calculations using density functional theory (DFT) as implemented in the ABINIT 8.2.2 package.[1,2] A cutoff energy of 1089 eV (40 hartrees) is used for the plane-wave basis set. The Brillouin zone integration is calculated with a Monkhorst-Pack[3] k point sampling of $12 \times 12 \times 8$ ($12 \times 12 \times 1$) for bulk (monolayer) h-MgO structures and $12 \times 12 \times 8$ for wurtzite structures. For monolayer structures, a 20 Å vacuum spacing ensures that the interaction between adjacent layers is negligible. For structural relaxations and phonon calculations, we employ the Perdew-Burke-Ernzerhof generalized gradient approximation (PBE-GGA)[4] and optimized norm-conserving Vanderbilt pseudopotentials.[5] The DFT-D3 dispersion correction[6] with Becke-Jonson (BJ) damping[7] is used to improve the description of long-range interaction. The dispersion contribution, typically essential for weakly bound layered materials (e.g., h-BN)[8] and molecular crystals, is included here not only for layered h-BN, but also for dense nonlayered h-MgO structures. In the latter case, the combination of GGA and the dispersion correction increases the accuracy of determining structural properties[9,10] and relative stability.[11] The same dispersion correction is also applied to monolayer structures for consistency.

For 1L and bulk h-BN and h-AlN, we obtain and present in fat band plots densities of states projected on the cation- and anion-centered s, p, and d orbitals identified by angular momentum and magnetic quantum numbers l and m. The atomic radii chosen for h-BN are 1.86 bohr for B and 1.83 bohr for N, and for h-AlN are 2.50 bohr for Al and 1.96 bohr for N. The sum projected density of states (PDOS) of a band state $|\psi\rangle$ onto both $|l, m\rangle = |1, \pm1\rangle$ is presented as the PDOS onto $p_{x,y}$ orbitals, because $|\langle 1,-1|\psi\rangle|^2 + |\langle 1,1|\psi\rangle|^2 = |\langle p_x|\psi\rangle|^2 + |\langle p_y|\psi\rangle|^2$.

Phonon dispersions are calculated using density-functional perturbation theory (DFPT)[12] with **q** point grids of $8 \times 8 \times 6$ and $8 \times 8 \times 1$ for bulk and isolated monolayer h-MgO type of structures, respectively. The relaxed GGA crystal structures are used in phonon calculations.

For more reliable band-gap prediction, the standard one-shot G_0W_0 approach[13,14] is applied on top of DFT calculations using local density approximation (LDA) and Fritz-Haber-Institute (FHI) pseudopotentials (the Troullier-Martins scheme).[15] Band structures are calculated based on the relaxed LDA crystal structures. Plasmon-pole approximation[14] is employed to evaluate the screened Coulomb interaction. The cutoff energy used to represent the dielectric matrix is set to 435 eV (16 hartrees). The number of bands is set to 200 for the screening and the self-energy calculations.

Electron energy-loss near-edge fine structures (ELNES) or x-ray absorption near-edge fine structures (XANES) which can reveal unoccupied electronic states are calculated by an extensively tested core-level spectra module within the CASTEP code.[16,17] The matrix elements are explicitly evaluated by the projector augmented wave (PAW) reconstruction method. The core-hole effect is treated by the specially constructed "excited" pseudopotential combined with the supercell approximation. A $3 \times 3 \times 2$ (72-atom) supercell is employed for h-BN and h-MgO type bulk crystals to isolate the excited centers in neighboring cells. A 3×3 planar layer and a 20 Å vacuum space isolating adjacent layers are used to construct the supercell for monolayer crystals. A Lorentzian broadening function with a full width at half maximum (FWHM) of 0.2 eV is used in Brillouin zone spectral integration and the spectra are further convolved by a Gaussian with an FWHM of 0.3 eV to mimic the effect of instrumental energy resolution.

Maximally localized Wannier functions are constructed using the Wannier90 code (version 2.1.0)[18] interfaced with PWSCF within Quantum ESPRESSO (version 6.3).[19] Only valence bands are selected in the construction of sp^2 and p_z Wannier orbitals. Antisymmetry about the atomic layer is imposed for p_z Wannier orbitals using the symmetry adapted mode.[20]

Literature on structural transitions upon ionicity increase induced by compression

It has been long known that the fourfold coordinated phases of $A^N B^{8-N}$ transition upon compression into higher coordination, higher ionicity ones following a general trend of decreasing equilibrium volume, [21-30] although loading triaxiality may be required.[21,23,27] If negative pressures can be introduced to achieve volume expansion, compounds that are thermodynamically stable in a high coordination polymorph (e.g., the NaCl structure, coordination number N_c = 6) at ambient pressure can transform into a low N_c form (e.g., wurtzite or zincblende, N_c = 4).[21,24] For some $A^N B^{8-N}$, an N_c = 5 intermediate phase is found along the transition path between the N_c = 4 and N_c = 6 ones.

This N_c = 5 structure has the same symmetry ($P6_3/mmc$) as h-BN. It has been referred to in the literature as the 5-5 structure since cation *A* and anion *B* are mutually fivefold coordinated,[24,29-32] or simply as HX standing for hexagonal.[23] Here we adopt the term h-MgO structure (just as NaCl for the rocksalt structure, the compound not necessarily MgO), after the first prediction of this structure.[21]

As the present work focuses on the bonding between its honeycomb atomic planes, we emphasize that the h-MgO structure is distinct from the layered, threefold coordinated h-BN structure characterized by weak van der Waals (vdW) interlayer interaction. Interestingly, h-BN transition into the wurtzite phase w-BN (while rhombohedral r-BN, another layered polymorph, morphs into zincblende z-BN, each transition preserving stacking sequence) upon compression,[28] consistent with the above trend.[21] An overall trend thus emerges: an octet $A^N B^{8-N}$ may transition along the path h-BN structure (N_c = 3) → wurtzite (N_c = 4) → h-MgO structure (N_c = 5) → rocksalt (N_c = 6) → CsCl structure (N_c = 8), with increasing ionicity upon successive compression,[21,24,28] if controlled with proper loading triaxiality.[21,23,27] The h-MgO phases, energetically stable only at negative pressures or experimentally challenging high pressures or loading triaxiality along this path, however, are metastable in ambient conditions for some compounds,[21,33] which are the focus of the present work; the trend described above serves to delineate the order of coordination and ionicity of the polymorphs.

Angle-resolved core-level spectroscopy

Under the dipole approximation,[34,35] the differential cross-section for momentum transfer **q** upon inelastic electron scattering is $|\langle i|\exp(i\mathbf{q}\cdot\mathbf{r})|f\rangle|^2 \approx |\mathbf{q}\cdot\langle i|\mathbf{r}|f\rangle|^2$, where **r** is the position vector, and $|i\rangle$ and $|f\rangle$ are respectively the initial atomic core state (1*s* for *K*-edge) and the final state (e.g., π^* or σ^* with the core hole), orthogonal to each other. The dependence of the spectra on the direction of **q** in angle-resolved ELNES therefore reveals the anisotropy and symmetry of orbital $|f\rangle$: The differential cross-sections for **q** // **c** and **q** ⊥ **c** originate from transitions to unoccupied states $|f_o\rangle$ and $|f_e\rangle$ with odd and even reflection symmetries with regard to the basal plane, respectively, since $\langle 1s|\mathbf{r}|f_o\rangle$ // **c** and $\langle 1s|\mathbf{r}|f_e\rangle \perp$ **c**. Thus, *K*-edge transitions to p_z- and $p_{x,y}$-like states ($|f_o\rangle$ and $|f_e\rangle$) dominate the angle-resolved spectra for **q** // **c** and **q** ⊥ **c**, respectively,[36,37] while transitions to *s*-like states are prohibited. These spectra can also be interpreted as X-ray absorption near-edge

structures (XANES) for electric field polarizations **E** // **c** and **E** $\perp$ **c**, due to the formally similar dipole matrix elements for electromagnetic radiation absorption.[35,38,39]

Therefore, angle-resolved near-edge spectra qualitatively (considering core-hole effects) represent densities of states projected onto p_z- and $p_{x,y}$-like symmetries, thus transition peaks are identified as originating from π^* (p_z) and σ^* ($p_{x,y}$) states.

For graphite and h-BN, the interlayer state contribution to core-level transition spectra is negligible due to small spatial overlap of these planar states, concentrated around the midplane between adjacent layers, with core states (e.g., 1*s* for *K* edge),[40] not interfering in the detection of σ^* and π^* peaks in core-level transition spectroscopies. For bulk h-AlN, the tighter space between (0001) honeycomb planes forces the free-electron-like "channel states" close to the atoms and sharing the space with the antibonding states. As the free-electron-like and antibonding states have comparable overlaps with core states, the free-electron-like states become detectable by core-level transition spectroscopies (if transition allowed). Moreover, the channel states in the band hosting the *s*-characterized parabolic CBM, squeezed into the same spatial region with the antibonding states, mix with these states and gain plenty of $p_{x,y}$ characters away from Γ (Fig. 6 of the main article), allowing $p_{x,y}$ transitions at lower energies than the lowest p_z transition peak.

Epitaxial h-MgO structured $A^N B^{8-N}$ ultrathin films on substrates

As discussed in the main text, p_z-π bonding in ***freestanding*** 1L h-MgO structured $A^N B^{8-N}$ is similar to that in h-BN, whereby anion (e.g. N in h-AlN) p_z orbitals donate electrons to the cation orbitals (Al-p_z). When a second monolayer is stacked onto the first to form a bilayer, the opposing N- and Al-p_z lobes overlap to form the vertical p_z bonds and forego in-plane π bonding. As a result, the Al-N bond length, and equivalently the basal plane lattice parameter, increase. Nevertheless, the outward pointing p_z lobes still interact with each other in a manner reminiscent of π bonding. Therefore, the basal-plane Al-N bond length and lattice parameter a_{2L} do not reach the bulk value a_h. As more and more MLs are stacked on, the lattice parameter a_{nL} increases with increasing layer count n to approach the bulk value a_h as the influence of the two terminating surfaces diminishes. Thus, the change from a_{1L} to a_{2L} is the steepest, followed by increasingly gradual changes, as shown by previous calculations[41] as well as our own (tables below).

As such, an energy barrier is expected in the pathway from 1L to 2L, due to the changes in bonding and concomitantly in lattice parameters. On the other hand, disturbance in the environment that nudges the anions and cations in opposite directions along the normal of the 1L will lead to mixing-in of sp^3 hybridization. Due to the close proximity of a_{1L} to a_w, the basal-plane lattice parameter of the wurtzite counterpart, the growth can more easily proceed towards wurtzite than through lateral expansion ($a_h > a_{1L}$) to retain the planar h-MgO structure, despite energetic preference for the latter.

The metal substrates in previous experiments provide the nudge. Investigations into h-BN on Ni(111) have shown that the p_z-electron-poor B is attracted to the metal surface while the p_z-electron-rich N repelled.[42-44] Similarly, we expect the electron-poor cation (Al, Mg, Zn) p_z orbitals to be more attracted to the metal surfaces than the electron-rich anion (N, O) p_z orbitals, as shown in Fig. S5, although buckling is not visible in all experiments discussed here on the Ag or Au surfaces, which are inert compared with Ni(111). The interaction with the substrate alters the bonding character of the 1L h-MgO structured $A^N B^{8-N}$ from the freestanding case.

On the one hand, the cation p_z lobes facing the metal surface, mixing with electrons in the metal, do not fully form π bonds with the anions. In this sense, this first monolayer is reminiscent of the top monolayer in a multilayer film. On the other hand, this interaction with the relatively inert noble metal surface is not sufficient to result in significant buckling (in first-principles calculations[41,45]) by countering the strong energetic preference towards planarity. Thus, the in-plane lattice parameter of the 1L h-MgO structured $A^N B^{8-N}$ on the Ag or Au(111) surface is close to the value for the freestanding monolayer. This situation is to be compared with the h-BN/Ni(111) system, where the 1L film buckling is observable and the growth of sp^3 coordinated zincblende BN can be induced.[43]

The preference that the cation is more attracted to the substrate surface, however, already exists in the first monolayer deposited on the metal surface. Then, the anion p_z in the second layer deposited (Fig. S5) does not see the cation p_z in the first layer as electron-poor as in the freestanding case and is thus not as attracted to it. A similar (yet opposite) argument can be made that the cation in the second monolayer is more attracted towards the anion in the first monolayer. As the growth proceeds layer by layer (Fig. S5), this tendency accumulates. To maintain planarity, the basal plane lattice would have to expand to approach the bulk value a_h determined by the length of the sp^2-σ bond without p_z-π bonding, with the influence of the π-like bonding of the outward pointing p_z lobes of the top monolayer diminishing. A lower-energy cost pathway, however, is buckling, which is an extension of the already-existing tendency for the cations and anions to reside in different planes, shifted towards and away from the substrate, respectively. Eventually, the system ends up with predominant sp^3 coordination, keeping the initial basal plane lattice parameter virtually unchanged ($a_{1L} \approx a_w$ with difference $\leq$ 0.7%). The initial planar 1L film thus smoothly and gradually transition into the wurtzite phase.

For ultrathin MgO on Ag(111),[41] we list below the experimental basal-plane lattice parameter values, along with calculated values by Goniakowski *et al.*[41] and in the present work, both for freestanding films:

Table S1. Experimental and calculated basal-plane lattice parameters for ultrathin MgO films.

	a_{1L} (Å)	a_{5L} (Å)	a_h (Å)	a_w (Å)
Experimental	3.25 $\pm$ 0.03	3.28 $\pm$ 0.03		
Goniakowski *et al.*[41]	3.26	3.43	3.49	
This work	3.294	3.431	3.486	3.296

Here, Table S1 (as do Tables S2 and S3 below) lists in-plane lattice parameter values of few-layer ultrathin films to facilitate direct comparison with experimental and prior computational results, in addition to those in Table 1 for free-standing 1L and bulk h-MgO type structures.

At the thickness of 5 ML, the measured a_{5L} does not match the value calculated by Goniakowski *et al.*, which is already close to the bulk values a_h calculated both by Goniakowski *et al.* and in this work. The measured a_{5L}, however, is close to the measured and calculated values of a_{1L}, and also to our calculated value a_w for the wurtzite phase, in line with the general picture discussed above. Notice that the relative difference between a_{1L} and a_w, $|(a_{1L} - a_w) / a_{1L}|$ = 0.0009, is particularly small for MgO. We therefore believe the ultrathin MgO film on Ag(111) assumes a wurtzite-like structure at thicknesses around 5 ML and above, even though bulk w-MgO is not thermodynamically stable under ambient (or zero pressure) conditions. Goniakowski *et al.* attributed the absence of the theoretically predicted evolutionary trend in lattice parameter to possible

stacking faults, which in calculations brought a_{5L} values closer to yet still larger than the observed, at a very high energy cost that renders h-MgO no longer “by far the most stable” phase with their presence.

In the following table, we list the experimental basal-plane lattice parameter values of ultrathin AlN on Ag(111),[46] along with calculated values by Bacaksiz *et al.*[33] and in this work:

Table S2. Experimental and calculated basal-plane lattice parameters for ultrathin AlN films.

	a_{1L} (Å)	a_{2L} (Å)	a_{4L} (Å)	a_h (Å)	a_w (Å)
Experimental[46]	3.14 ± 0.06		3.13		
Bacaksiz *et al.*[33]	3.13	3.20		3.30	3.11
This work	3.123	3.194	3.236	3.294	3.108

Just as the above case of ultrathin MgO on Ag(111), the ultrathin AlN on Ag(111) fails to show the predicted structural evolution despite excellent agreement with theory in a_{1L}. At the thickness of 4 ML, the basal-plane lattice parameter virtually remains unchanged from a_{1L}, which is close to the wurtzite value a_w. For AlN, the calculated relative difference $(a_{1L} - a_w) / a_{1L} = 0.005$.

In the following, we compare the experimental results of ultrathin ZnO on Ag(111)[47] and Au(111)[45] with calculations with the Au substrate considered and of freestanding h-ZnO in this work.

Table S3. Experimental and calculated basal-plane lattice parameters for ultrathin ZnO films.

	a_{1L} (Å)	a_{2L} (Å)	a_{3L} (Å)	a_{4L} (Å)	a_h (Å)	a_w (Å)
ZnO/Ag(111)[47]			3.303			3.249[a]
ZnO/Au(111)[45]	3.29±0.02	3.35	3.325	3.24		3.25[a]
ZnO/Au(111) calculated[45]	3.30	3.36	3.38	3.36		
Freestanding, this work	3.275	3.316	3.352	3.366	3.427	3.251

[a] Experimental value cited by but not measured in the referenced works.

Tusche *et al.*[47] measured a thickness independent basal-plane lattice parameter of 3.303 Å for ultrathin ZnO on Ag(111) with average thicknesses of 2.7 and 3.5 ML, both films grown in a three-dimensional mode with regions 2 to 4 ML thick. More important, they found the fourth monolayer to be wurtzite-like. Lee *et al.*[45] observed an increase from a_{1L} to a_{2L} for ZnO on Au(111) but then the basal-plane lattice parameter started to decrease with increasing thickness, reaching the wurtzite value a_w at a thickness of 4 ML. Interestingly, their first-principles calculation considering the Au substrate partially accounted for this trend, although much slower than the observed. The discrepancy is likely caused by the model setup where a small ZnO island is placed on Au(111) to handle incommensurate ZnO and Au lattices. Moreover, even though not fully accounting for the evolution from a_{1L} to a_{4L}, their calculation revealed “very small corrugation” in ZnO on Au(111) “with a preference for O-termination”,[45] consistent with the tendency for the cation to shift to towards the metal substrate as we inferred from experimental observations of h-BN on Ni(111).

All these cases suggest that ultrathin $A^N B^{8-N}$ films on noble metal surfaces with planar bottom monolayers evolve into the wurtzite structure as more layers are deposited. This salient common behavior in previous

experiments is facilely explained within the unusual bonding picture of the h-MgO structure phase of these binary octet compounds.

References

1 X. Gonze, B. Amadon, P. M. Anglade, J. M. Beuken, F. Bottin, P. Boulanger, F. Bruneval, D. Caliste, R. Caracas and M. Côté, *Comput. Phys. Commun.*, 2009, **180**, 2582-2615.

2 X. Gonze, F. Jollet, F. A. Araujo, D. Adams, B. Amadon, T. Applencourt, C. Audouze, J. M. Beuken, J. Bieder, A. Bokhanchuk, E. Bousquet, F. Bruneval, D. Caliste, M. Cote, F. Dahm, F. Da Pieve, M. Delaveau, M. Di Gennaro, B. Dorado, C. Espejo, G. Geneste, L. Genovese, A. Gerossier, M. Giantomassi, Y. Gillet, D. R. Hamann, L. He, G. Jomard, J. L. Janssen, S. Le Roux, A. Levitt, A. Lherbier, F. Liu, I. Lukacevic, A. Martin, C. Martins, M. J. T. Oliveira, S. Ponce, Y. Pouillon, T. Rangel, G. M. Rignanese, A. H. Romero, B. Rousseau, O. Rubel, A. A. Shukri, M. Stankovski, M. Torrent, M. J. Van Setten, B. Van Troeye, M. J. Verstraete, D. Waroquiers, J. Wiktor, B. Xu, A. Zhou and J. W. Zwanziger, *Comput. Phys. Commun.*, 2016, **205**, 106-131.

3 H. J. Monkhorst and J. D. Pack, *Phys. Rev. B*, 1976, **13**, 5188-5192.

4 J. P. Perdew, K. Burke and M. Ernzerhof, *Phys. Rev. Lett.*, 1996, **77**, 3865-3868.

5 D. R. Hamann, *Phys. Rev. B*, 2013, **88**, 085117.

6 L. Goerigk and S. Grimme, *Phys. Chem. Chem. Phys.*, 2011, **13**, 6670-6688.

7 A. D. Becke and E. R. Johnson, *J. Chem. Phys.*, 2006, **124**, 221101.

8 S.-P. Gao, *Solid State Communications*, 2012, **152**, 1817-1820.

9 A. Sun, S.-P. Gao and G. Gu, *Phys. Rev. Mater.*, 2019, **3**, 104604.

10 F. Ortmann, F. Bechstedt and W. G. Schmidt, *Phys. Rev. B*, 2006, **73**, 205101.

11 M. Halo, C. Pisani, L. Maschio, S. Casassa, M. Schütz and D. Usvyat, *Phys. Rev. B*, 2011, **83**, 035117.

12 X. Gonze and C. Lee, *Phys. Rev. B*, 1997, **55**, 10355-10368.

13 G. Onida, L. Reining and A. Rubio, *Rev. Mod. Phys.*, 2002, **74**, 601-659.

14 R. W. Godby and R. J. Needs, *Phys. Rev. Lett.*, 1989, **62**, 1169-1172.

15 M. Fuchs and M. Scheffler, *Comput. Phys. Commun.*, 1999, **119**, 67-98.

16 S.-P. Gao, C. J. Pickard, M. C. Payne, J. Zhu and J. Yuan, *Phys. Rev. B*, 2008, **77**, 115122.

17 S.-P. Gao, C. J. Pickard, A. Perlov and V. Milman, *J. Phys. Condens. Matter*, 2009, **21**, 104203.

18 A. A. Mostofi, J. R. Yates, G. Pizzi, Y.-S. Lee, I. Souza, D. Vanderbilt and N. Marzari, *Comput. Phys. Commun.*, 2014, **185**, 2309-2310.

19 P. Giannozzi, O. Andreussi, T. Brumme, O. Bunau, M. B. Nardelli, M. Calandra, R. Car, C. Cavazzoni, D. Ceresoli and M. Cococcioni, *J. Phys. Condens. Matter*, 2017, **29**, 465901.

20 R. Sakuma, *Phys. Rev. B*, 2013, **87**, 235109.

21 S. Limpijumnong and W. R. L. Lambrecht, *Phys. Rev. B*, 2001, **63**, 104103.

22 N. E. Christensen, S. Satpathy and Z. Pawlowska, *Phys. Rev. B*, 1987, **36**, 1032.

23 K. Sarasamak, A. J. Kulkarni, M. Zhou and S. Limpijumnong, *Phys. Rev. B*, 2008, **77**, 024104.

24 D. Zagorac, K. Doll, J. Zagorac, D. Jordanov and B. Matovic, *Inorg. Chem.*, 2017, **56**, 10644-10654.

25 J. C. Schön, *Z. Anorg. Allg. Chem.*, 2004, **630**, 2354-2366.

26 Ž. P. Čančarević, J. C. Schoen and M. Jansen, *Chem. Asian J.*, 2008, **3**, 561-572.

27 D. Wu, M. G. Lagally and F. Liu, *Phys. Rev. Lett.*, 2011, **107**, 236101.

28 R. M. Wentzcovitch, S. Fahy, M. L. Cohen and S. G. Louie, *Phys. Rev. B*, 1988, **38**, 6191-6195.
29 J. Zagorac, D. Zagorac, M. Rosić, J. C. Schön and B. Matović, *CrystEngComm*, 2017, **19**, 5259-5268.
30 J. Zagorac, D. Zagorac, D. Jovanović, J. Luković and B. Matović, *J. Phys. Chem. Solids*, 2018, **122**, 94-103.
31 D. Zagorac, J. C. Schön and M. Jansen, *Process. Appl. Ceram*, 2013, **7**, 37-41.
32 D. Zagorac, J. C. Schön, J. Zagorac and M. Jansen, *Phys. Rev. B*, 2014, **89**, 075201.
33 C. Bacaksiz, H. Sahin, H. D. Ozaydin, S. Horzum, R. T. Senger and F. M. Peeters, *Phys. Rev. B*, 2015, **91**, 085430.
34 M. Inokuti, *Rev. Mod. Phys.*, 1971, **43**, 297.
35 R. D. Leapman, P. L. Fejes and J. Silcox, *Phys. Rev. B*, 1983, **28**, 2361.
36 S.-P. Gao, A. Zhang, J. Zhu and J. Yuan, *Appl. Phys. Lett.*, 2004, **84**, 2784-2786.
37 J. Lu, S.-P. Gao and J. Yuan, *Ultramicroscopy*, 2012, **112**, 61-68.
38 S.-P. Gao, J. Zhu and J. Yuan, *Chem. Phys. Lett.*, 2004, **400**, 413-418.
39 J. Wang, Z. Wang, H. Cho, M. J. Kim, T. K. Sham and X. Sun, *Nanoscale*, 2015, **7**, 1718-1724.
40 M. Posternak, A. Baldereschi, A. J. Freeman, E. Wimmer and M. Weinert, *Phys. Rev. Lett.*, 1983, **50**, 761.
41 J. Goniakowski, C. Noguera and L. Giordano, *Phys. Rev. Lett.*, 2004, **93**, 215702.
42 R. Laskowski, P. Blaha and K. Schwarz, *Phys. Rev. B*, 2008, **78**, 045409.
43 E. Rokuta, Y. Hasegawa, K. Suzuki, Y. Gamou, C. Oshima and A. Nagashima, *Phys. Rev. Lett.*, 1997, **79**, 4609.
44 W. Auwärter, T. J. Kreutz, T. Greber and J. Osterwalder, *Surf. Sci.*, 1999, **429**, 229-236.
45 J. Lee, D. C. Sorescu and X. Deng, *J. Phys. Chem. Lett.*, 2016, **7**, 1335-1340.
46 P. Tsipas, S. Kassavetis, D. Tsoutsou, E. Xenogiannopoulou, E. Golias, S. A. Giamini, C. Grazianetti, D. Chiappe, A. Molle and M. Fanciulli, *Appl. Phys. Lett.*, 2013, **103**, 251605.
47 C. Tusche, H. L. Meyerheim and J. Kirschner, *Phys. Rev. Lett.*, 2007, **99**, 026102.
